# How low can we go? The effect of acquisition duration on cardiac volume and function measurements in free-running cardiac and respiratory motion-resolved five-dimensional whole-heart cine magnetic resonance imaging at 1.5T

**DOI:** 10.1016/j.jocmr.2025.101863

**Published:** 2025-02-14

**Authors:** Robert J. Holtackers, Augustin C. Ogier, Ludovica Romanin, Estelle Tenisch, Isabel Montón Quesada, Ruud B. van Heeswijk, Christopher W. Roy, Jérôme Yerly, Milan Prsa, Matthias Stuber

**Affiliations:** a Department of Radiology & Nuclear Medicine, Maastricht University Medical Center, Maastricht, the Netherlands; b Cardiovascular Research Institute Maastricht (CARIM), Maastricht University, Maastricht, the Netherlands; c Department of Radiology, Lausanne University Hospital (CHUV) and University of Lausanne (UNIL), Lausanne, Switzerland; d Advanced Clinical Imaging Technology, Siemens Healthineers International AG, Lausanne, Switzerland; e Center for Biomedical Imaging (CIBM), Lausanne, Switzerland; f Division of Pediatric Cardiology, Woman-Mother-Child Department, Lausanne University Hospital (CHUV) and University of Lausanne (UNIL), Lausanne, Switzerland

**Keywords:** CMR, Cardiac MRI, 5D, Free-running, Self-gating, Free-breathing

## Abstract

**Background:**

Cardiovascular magnetic resonance (CMR) is the gold standard for assessing cardiac volumes and function using two-dimensional (2D) breath-held cine imaging. This technique, however, requires a reliable electrocardiogram (ECG) signal, repetitive breath-holds, and the time-consuming and proficiency-demanding planning of cardiac views. Recently, a free-running framework has been developed for cardiac and respiratory motion-resolved five-dimensional (5D) whole-heart imaging without the need for an ECG signal, repetitive breath-holds, and meticulous plan scanning. In this study, we investigate the impact of acquisition time on cardiac volumetric and functional measurements, when using free-running imaging, compared to reference standard 2D cine imaging.

**Methods:**

Sixteen healthy adult volunteers underwent CMR at 1.5T, including standard 2D breath-held cine imaging and free-running imaging using acquisition durations ranging from 1 to 6 min in randomized order. All datasets were anonymized and analyzed for left-ventricular end-systolic volume (ESV) and end-diastolic volume (EDV), as well as ejection fraction (EF). In a subset of data, intra- and inter-observer agreement was assessed. In addition, image quality and observer confidence were scored using a 4-point Likert scale. Finally, acquisition efficiency was reported for both imaging techniques, which was defined as the time required for data sampling divided by the total scan time.

**Results:**

No significant differences in left-ventricular EDV and ESV were found between free-running imaging for 1, 2, 3, 5, and 6 min and standard 2D breath-held cine imaging. Biases in EDV ranged from −2.4 to −7.4 mL, while biases in ESV ranged from −3.8 to 2.1 mL. No significant differences in EF were found between free-running imaging of any acquisition duration and standard 2D breath-held cine imaging. Biases in EF ranged from −2.8% to 0.94%. Both image quality and observer confidence in free-running imaging improved when the acquisition duration increased. However, they were always lower than standard 2D breath-held cine imaging. Acquisition efficiency improved from 13% for standard 2D cine imaging to 50% or higher for free-running imaging.

**Conclusion:**

Free-running CMR with an acquisition duration as short as 1 min can provide left-ventricular cardiac volumes and EF comparable to standard 2D breath-held cine imaging, albeit at the expense of both image quality and observer confidence.

## Graphical abstract

**Figure:**
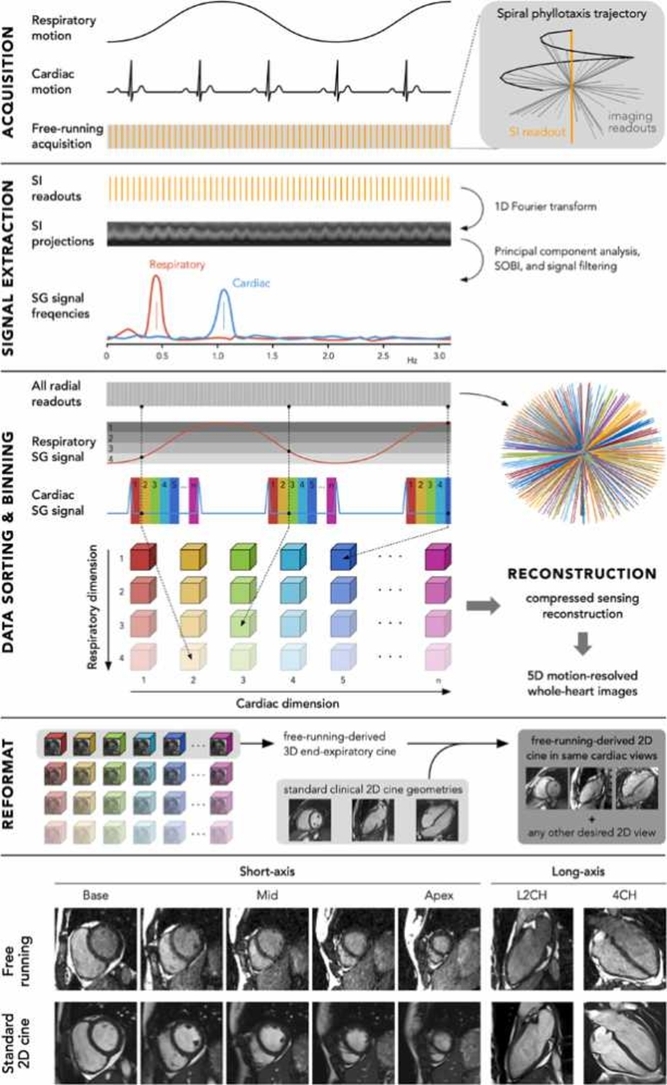

## 1 Introduction

Cardiovascular magnetic resonance (CMR) is the current reference standard for the quantification and assessment of cardiac volumes and function and is typically performed using two-dimensional (2D) balanced steady-state free precession (bSSFP) cine images [1], [2], [3]. In a typical clinical routine setting, cine images are acquired with an in-plane resolution between 1.0 and 2.0 mm and a slice thickness of 5–8 mm in various cardiac views using repetitive breath-holds of 10–15 s each [1]. Although 2D breath-held cine imaging is well-established, this technique has significant drawbacks.

First, typically 12–15 short-axis and 3 long-axis slices are required for an accurate cardiac functional assessment, requiring patients to hold their breath repetitively. Second, a slice gap is regularly introduced to reduce the number of slices and thereby breath-holds, which comes at the expense of reduced volumetric coverage of the heart. Finally, all the cardiac views need to be planned beforehand and no additional cardiac views can be reconstructed afterward. This planning is not only time consuming and requires significant expertise, but also requires additional breath-holds with interspersed respiratory recovery periods for the patient. As an alternative, three-dimensional (3D) cardiac cine imaging provides whole-heart coverage, simplifies planning, and allows for an improved signal-to-noise ratio (SNR) [4]. However, electrocardiogram (ECG) gating and navigator-gated acquisitions are still required to compensate for cardiac and respiratory motion. These affect setup time, workflow, and ease of use, and are thereby inefficient.

In the past 10 years, a free-running framework has been developed for simultaneous cardiac and respiratory motion-resolved five-dimensional (5D) whole-heart imaging with high isotropic spatial resolution (1.0–2.0 mm^3^) and high temporal resolution (15–30 phases per cardiac cycle) [5]. In this fully self-gated approach, both cardiac and respiratory signals are extracted from the raw data and compressed sensing (CS) is used for the reconstruction of 5D (x-y-z-cardiac-respiratory) images [6]. Without any cardiac view planning during acquisition, this framework allows for reconstruction of cine images in any desired cardiac view retrospectively and requires no CMR planning expertise. Due to its self-gating abilities, no ECG signal is required, and the patient can breathe freely.

Since boundaries of efficiency and simplicity of this free-running framework as an automated push-button solution have never been explored, the goal of the present work was to investigate the effect of acquisition time on the calculated left-ventricular cardiac volumes and function measurements, using standard 2D breath-held cine imaging as reference standard.

## 2 Methods

### 2.1 Study population

Sixteen adult volunteers without known cardiovascular disease were included and underwent CMR using a 1.5T whole-body clinical MR system (MAGNETOM Sola, Siemens Healthineers, Erlangen, Germany) with a standard 34 channel chest-spine receiver coil array of which the 24 elements closest to the field of view (FOV) were used. The study was approved by the local ethics committee (Ethics Committee of the Canton of Vaud (CER-VD) #2021-02458) and all patients provided written informed consent.

### 2.2 Standard 2D cine imaging

All study subjects underwent standard 2D cine imaging using a conventional ECG-gated bSSFP sequence in two-chamber, four-chamber, and a stack of short-axis planes. Typical sequence parameters were echo time (TE) = 1.4 ms, repetition time (TR) = 3.2 ms, radiofrequency (RF) excitation angle 80°, 1.4 × 1.4 mm^2^ in-plane resolution, 8 mm slice thickness, 2 mm slice gap, CS factor 7.5, no fat suppression, and a receiver bandwidth of 977 Hz/pixel. All 2D cine images were acquired using repetitive end-expiratory breath-holds of approximately 10 s, interspersed respiratory recovery periods of 15 s, and were reconstructed to 25 cardiac phases according to clinical protocol.

### 2.3 Free-running imaging

Following standard 2D cine imaging, free-running data were acquired during free breathing using a slab-selective 3D radial sequence with a spiral phyllotaxis bSSFP readout, without ECG gating or respiratory navigators [6]. Typical sequence parameters were TE = 1.74 ms, TR = 3.53 ms, FOV 220 × 220 × 220 mm^3^, 1.4 mm isotropic resolution, no fat suppression, receiver bandwidth 1116 Hz/pixel, and 22 radial signal readouts per spiral interleave. For each volunteer, the maximum allowed RF excitation angle was chosen (median 70°, range 58–70°). At the start of each spiral interleave (every ∼80 ms), a readout along the superior-inferior direction was acquired as a self-gating signal. With a temporal resolution of 80 ms, this self-gating signal allows for the extraction of cardiac and respiratory self-gating frequencies up to approximately 6 Hz, corresponding to a maximum detectable heart rate and respiratory rate of 360 beats/breaths per minute. The described free-running imaging was repeated six times, with uninterrupted acquisition durations ranging from 1 to 6 min, performed in randomized order to avoid confounding fatigue effects. Apart from the number of spiral interleaves (762 for 0:59 min, 1516 for 1:58 min, 2322 for 3:00 min, 3058 for 3:58 min, 3833 for 4:58 min, and 4621 for 5:59 min), all sequence parameters remained identical for these six acquisitions. Finally, acquisition efficiency was reported for both imaging techniques, which was defined as the time required for data sampling divided by the total scanner time for each technique.

### 2.4 Free-running data reconstruction

All raw imaging data were processed using a previously reported fully automated motion extraction and reconstruction framework in MATLAB (The Mathworks, Natick, Massachusetts, USA) [6]. Using principal component analysis and second-order blind identification (SOBI) [7], [8] of the self-gating signal, i.e. the superior-inferior readouts, the main respiratory and cardiac frequency were derived, and cardiac triggers and respiratory motion signal were extracted. Based on these extracted cardiac triggers, the imaging data were retrospectively sorted into cardiac phases with a bin width of 50 ms, which is deemed sufficient for healthy volunteers. Note that this cardiac bin width can be freely adjusted after acquisition to control the cardiac temporal resolution. The same imaging data were also divided into four equally populated respiratory motion states. As a result, all individual radial readouts were assigned to their respective cardiac and respiratory bin independently from their position in the spiral phyllotaxis interleave to maximize the reconstructed temporal resolution. Finally, a CS algorithm that exploits sparsity along both the cardiac and respiratory dimensions was used to reconstruct 5D (x-y-z-cardiac-respiratory) motion-resolved images. An alternating direction method of multipliers algorithm was used with a total of ten iterations to solve the CS optimization problem. The regularization weights along both the cardiac and respiratory dimensions were conservatively set to 0.001 for all acquisition times to avoid compression artefacts while minimalizing residual aliasing.

After the reconstruction of each free-running dataset, a 3D (x-y-z) image volume was available for each combination of cardiac phase and respiratory motion state. By combining the 3D volumes of all cardiac phases for the end-expiratory motion state, an end-expiratory 3D cine image was created. Using the slice orientation of the obtained standard 2D clinical cine images, each free-running 3D cine image was reformatted into 2D cine images in the exact same short-axis and long-axis two- and four-chamber cardiac views.

**Fig. 1:**
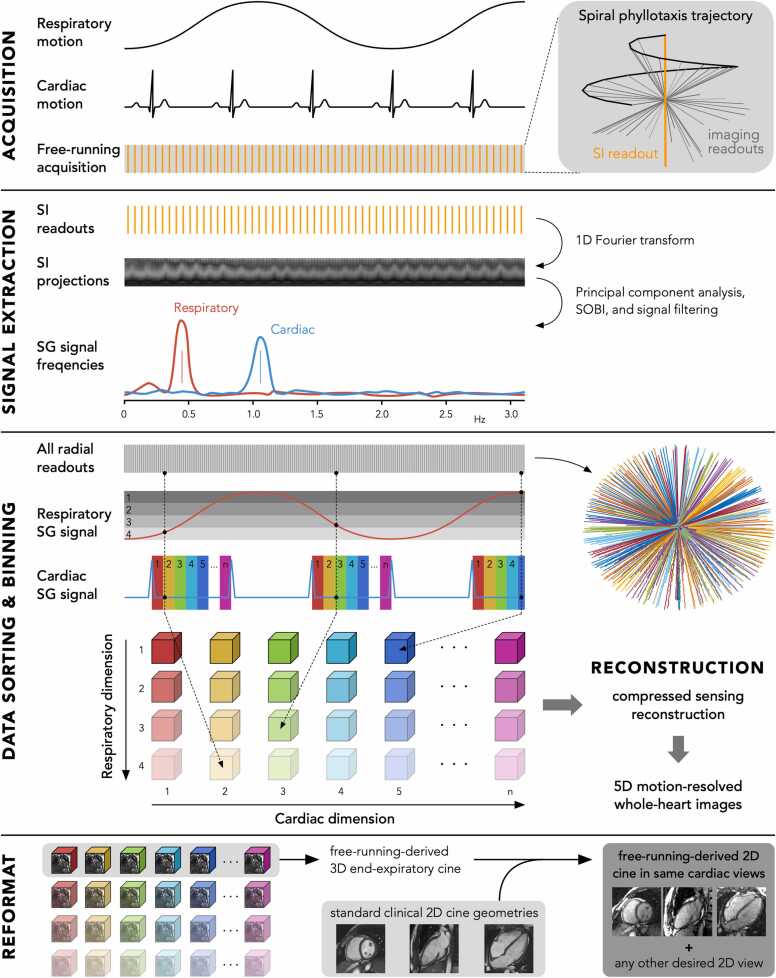
Schematic overview of the free-running framework. This framework consists of a 1) continuous 3D radial acquisition with a spiral phyllotaxis readout, 2) self-gating signal extraction using principal component analysis and SOBI, 3) data sorting and binning using the cardiac and respiratory self-gating signals, 4) CS reconstruction, and 5) data reformatting using the standard clinical 2D cine geometries. *SOBI* second-order blind identification, *SG* self-gated, *SI* superior-inferior, *CS* compressed sensing, *2D* two-dimensional, *3D*, three-dimensional

The entire above-mentioned reconstruction and reformatting of the acquired free-running data was performed using a single mouse click without any further interaction. A graphical overview of the entire free-running framework is shown in Fig. 1.

### 2.5 Image analysis

For each of the 16 volunteers, 1 set of standard 2D breath-held cine and 6 sets of free-running-derived cine images were available, resulting in 112 datasets, each containing a stack of short-axis and both 2- and 4-chamber long-axis cine images. All 112 datasets were anonymized, randomized, and analyzed by an expert reader with over 10 years of experience in CMR (M.P.) using the cvi42 (Circle Cardiovascular Imaging, Calgary, Alberta, Canada) software. For each dataset, the end-systolic and end-diastolic phases were selected, followed by manual segmentation of the left-ventricular blood pool in all short-axis slices for both cardiac phases [9]. The left-ventricular end-systolic volume (ESV), end-diastolic volume (EDV), and ejection function (EF) were then calculated. In a subset of five volunteers, the quantitative measures were assessed again by a second observer (E.T.) to evaluate both intra- and inter-observer agreement.

In addition to the quantitative analysis, each dataset was rated using a 4-point Likert scale for its image quality and observer confidence in segmentation. Overall image quality was rated as poor, moderate, good, or excellent, while observer confidence in segmentation was rated as not confident at all, slightly confident, fairly confident, or completely confident.

### 2.6 Statistical analysis

Cardiac volumes and ejection fractions (EFs) derived from the free-running acquisitions were compared to those derived from reference standard 2D breath-held cine using either a paired-sample Student’s *t*-test (normally distributed data) or a non-parametric Wilcoxon signed-rank test (non-normally distributed data). Normality of data distribution was evaluated using the Shapiro–Wilk test. Bland–Altman analysis was used to evaluate agreement between the EFs derived from free-running cine and standard 2D breath-held cine. The Likert scale scores for both qualitative assessments (image quality and observer confidence) were evaluated using the non-parametric Wilcoxon signed-rank test. Intra- and inter-observer agreements were assessed for all qualitative measures using the intraclass correlation coefficient (ICC, two-way mixed model for absolute agreement). ICCs were classified as poor (<0.40), moderate (0.40–0.60), good (0.60–0.80), and excellent (>0.80). All statistical analyses were performed using the Statistical Package for the Social Sciences (SPSS, International Business Machines, Armonk, New York, USA). All statistical tests were two-tailed and p-values <0.05 were considered significant. Results are expressed as mean ±standard deviation or as percentage unless specified otherwise.

## 3 Results

**Fig. 2:**
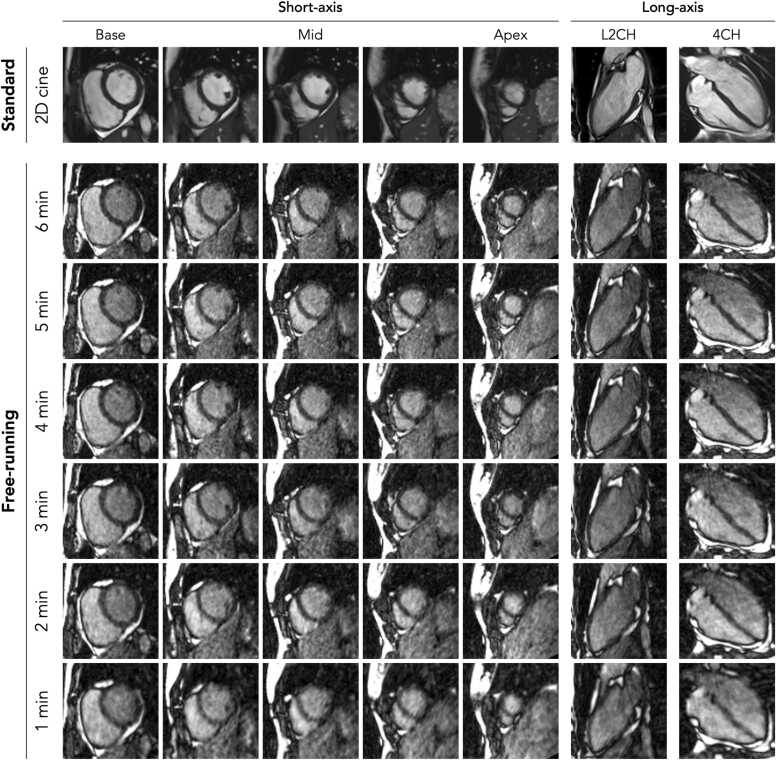
Image examples acquired using reference standard 2D breath-held cine imaging and as derived from the 3D free-running acquisitions using different scan durations (1 to 6 min). Various short-axis (from base to apex) and long-axis (both left-ventricular two-chamber and four-chamber) cardiac views are shown, all in the end-diastolic phase of the cardiac cycle. Note that the 2D cine images have an 8 mm slice thickness compared to the 1.4 mm reformats of the free-running cine images. *2D* two-dimensional, *3D*, three-dimensional

Among the 16 healthy subjects, 8 were female (50%), and the median age was 24 years (range 19–30). Both reference standard 2D breath-held cine images and free-running data with six different scan durations were successfully acquired in all subjects (Fig. 2).

### 3.1 Quantitative analysis

All quantitative results of the volumetric and functional analysis can be found in Table 1. No significant differences in mean left-ventricular EDV and ESV were found between free-running imaging for 1, 2, 3, 5, and 6 min and standard 2D breath-held cine imaging. Although free-running imaging for 4 min also showed no significant difference in mean ESV, a statistically significant average underestimation in mean EDV of 7 mL (p = 0.026) was found. Biases in EDV ranged from −2.4 to −7.4 mL between free-running imaging and reference standard 2D breath-held cine imaging, while biases in ESV ranged from −3.8 to 2.1 mL.

**Table 1:** Overview of the quantitative analysis.

	EDV	ESV	EF	EDV bias	LOA	ESV bias	LOA	EF bias	LOA
2D cine	159±33	62±15	61±4	Compared to 2D cine		Compared to 2D cine		Compared to 2D cine	
FR 6 min	156±35	63±16	60±4	−2±13	−28 to 23	1±7	−13 to −14	−1±4	−9 to 7
FR 5 min	154±35	64±17	58±5	−5±14	−33 to 23	2±9	−15 to 20	−3±6	−15 to 9
FR 4 min	151±33	61±16	60±4	−7±12	−31 to 16	−1±7	−14 to −12	−1±5	−10 to 8
FR 3 min	154±33	63±18	60±6	−5±16	−36 to 27	1±9	−17 to 18	−1±6	−13 to 10
FR 2 min	153±33	61±18	61±4	−6±16	−38 to 26	−1±10	−21 to 19	0±5	−10 to 9
FR 1 min	155±31	58±12	62±4	−4±11	−26 to 17	−4±8	−20 to 13	1±5	−9 to 10

The shown biases indicate the difference between the indicated free-running acquisition and standard 2D cine. All volumes are in mL, EF is in %. *FR* free-running, *EDV* end-diastolic volume, *ESV* end-systolic volume, *EF* ejection fraction, *LOA* limits of agreement, *2D* two-dimensional

**Fig. 3:**
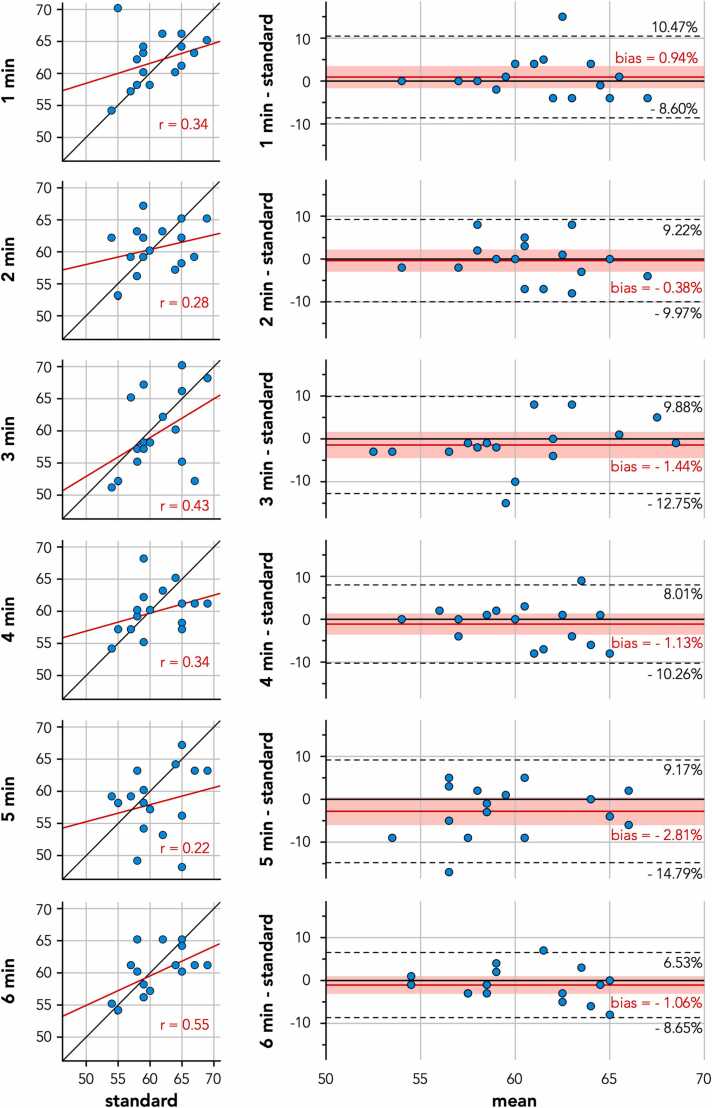
Correlation plots and Bland–Altman analysis of the left-ventricular ejection fractions as derived from free-running cine using various durations (1–6 min) compared to reference standard 2D breath-held cine imaging. The solid red line and red shaded area in each Bland–Altman plot indicate the bias with its 95% confidence interval, while the black dotted lines indicate the limits of agreement. All axes indicate absolute percentages. *2D* two-dimensional

No significant differences in mean EF were found between standard free-running imaging using any acquisition duration and standard 2D breath-held cine. Bland–Altman analysis showed biases in EF ranging from −2.8% to 0.94% between free-running imaging and reference standard 2D breath-hold cine imaging (Fig. 3). Free-running imaging for 6 min showed the narrowest limits of agreement of <10% (−8.65–6.53%).

### 3.2 Intra- and inter-observer agreement of the quantitative analysis

The results of the intra- and inter-observer agreement for all quantitative measures can be found in Table 2. Excellent intra-observer agreement in EF measurements was found for both standard 2D cine (ICC 0.946) and 1, 3, and 5 min of free-running imaging (ICC 0.823, 0.909, and 0.873, respectively). Good agreement was found for 4 and 6 min (ICC 0.620 and 0.761, respectively) of free-running, while poor agreement was found for 2 min (ICC 0.355) of free-running. The relative intra-observer difference in EF measurements for standard 2D cine was −2%, which was similar to 1, 3, and 5 min of free-running. Free-running for 2, 4, and 6 min showed relative differences of 5, 2, and −3%, respectively.

**Table 2:** Intra- and inter-observer agreement of the quantitative analysis.

	Intra-observer						Inter-observer					
	ICC			Rel. difference [%]			ICC			Rel. difference [%]		
	EDV	ESV	EF	EDV	ESV	EF	EDV	ESV	EF	EDV	ESV	EF
2D cine	0.996	0.983	0.946	0	3	−2	0.850	0.547	0.123	12	28	−8
FR 6 min	0.991	0.863	0.761	−1	3	−3	0.671	0.368	0.438	15	23	−5
FR 5 min	0.991	0.845	0.873	0	4	−2	0.813	0.390	0.478	7	23	−10
FR 4 min	0.996	0.869	0.620	−1	−3	2	0.711	0.476	0.094	13	24	−6
FR 3 min	0.956	0.980	0.909	−3	1	−2	0.719	0.044	−0.598	13	28	−6
FR 2 min	0.951	0.685	0.355	−2	−9	5	0.490	0.277	0.251	16	26	−5
FR 1 min	0.976	0.944	0.823	−3	0	−2	0.711	0.262	−0.052	13	31	−8

For both intra-observer and inter-observer agreement, the intraclass correlation coefficients and relative (rel.) differences (i.e., the average differences between the two observations as a percentage of the initial observation) are shown. .*ICC* intraclass correlation coefficient, *FR* free-running*, EDV* end-diastolic volume*, ESV* end-systolic volume*, EF* ejection fraction, *2D* two-dimensional

Poor inter-observer agreement in EF measurements was found for both standard 2D cine and free-running imaging for 1, 2, 3, and 4 min (ICC −0.052, 0.251, −0.598, and 0.094, respectively). Moderate inter-observer agreement was found for free-running imaging for 5 and 6 min (ICC 0.478 and 0.438, respectively). The relative inter-observer difference in EF measurements for standard 2D cine was −8%, while the relative differences for free-running imaging varied between −5% (2 min) and −10% (5 min).

### 3.3 Qualitative analysis

**Fig. 4:**
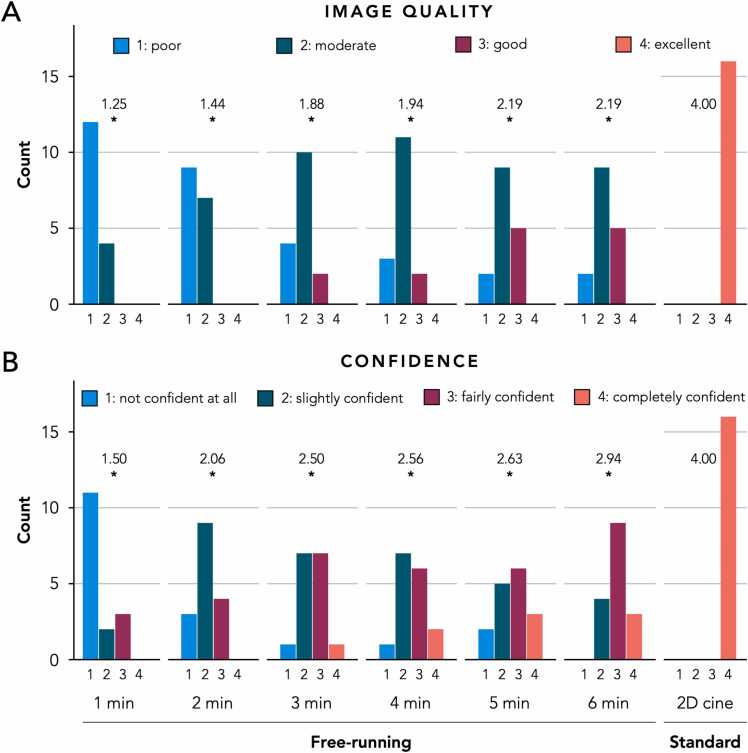
Bar plots indicating the Likert scale score frequencies for both overall image quality (upper panel) and observer confidence in segmentation (lower panel) for free-running cine imaging using various acquisition durations (1 to 6 min) and reference standard 2D cine imaging. For each type of acquisition, the average score is indicated. The asterisks indicate a significant difference compared to reference standard 2D cine imaging. *2D* two-dimensional

Image quality for free-running imaging improved when the acquisition duration increased, showing a mean score of 1.25 (interquartile range (IQR) 1.00–1.75) for the 1-min acquisition and 2.19 (IQR 2.00–3.00) for both the 5- and 6-min acquisitions. The observer confidence in segmentation for free-running imaging also improved with increasing acquisition duration, showing a mean score of 1.50 (IQR 1.00–2.00) for the 1-min acquisition and 2.94 (IQR 2.25–3.00) for the 6-min acquisition. For both image quality and observer confidence, however, standard 2D breath-held cine imaging showed a significantly higher score compared to free-running imaging using any acquisition duration (Fig. 4).

### 3.4 Scan time and acquisition efficiency

The average time required for the entire planning and acquisition of 2D breath-held cine imaging in both short-axis and long-axis views, including the preceding localizer images, was 14:29 min (range 11:02–18:00). On average, during 1:52 min (range 1:13–2:43) of that time MRI data were acquired, resulting in an average acquisition efficiency of 12.9% (range 9.1–20.0%). For free-running imaging no cardiac views need to be planned, thereby the 3D acquisition and shim volumes were planned under 1 min and used identically for all acquisition durations from 1 to 6 min. The average time required for planning and acquisition of free-running imaging ranged from maximum 2 min for the 1-min acquisition to maximum 7 min for the 6-min acquisition. Acquisition efficiency thereby ranged from approximately 50% for the 1-min acquisition to 85.7% for the 6-min acquisition.

## 4 Discussion

In the present study, we compared cardiac free-running cine imaging with varying acquisition durations, ranging from 1 to 6 minutes, against reference standard 2D breath-held cine imaging. The study findings show that free-running cine imaging with acquisition durations as short as 1-minute leads to EFs comparable to those acquired using reference standard 2D breath-held cine imaging.

Free-running CMR has significant advantages over standard 2D breath-held imaging. Standard cine imaging requires a reliable ECG signal for retrospective gating to “freeze” cardiac motion and repetitive breath-holds to eliminate respiratory motion [1]. When failing to meet both requirements adequately, the resulting images can be blurred and are possibly not suitable for diagnosis. Accurate R-wave detection in the ECG recording is often hampered due to unwanted overlaid signals from the magnetohydrodynamic effect and rapid gradient switching, particularly in uninterrupted acquisition such as during cine imaging [10]. In addition, repetitive breath-holds can cause discomfort and fatigue, or may not even be possible for the patient. Furthermore, consecutive breath-holds can be inconsistent, with the resultant slices actually overlapping, introducing measurement errors. Free-running CMR, on the other hand, uses a self-gating sequence that can extract the cardiac and respiratory motion signal directly from the acquired image data, thereby obviating the need for an ECG signal and repetitive breath-holds [5], [6].

In addition, standard 2D cine imaging requires the time-consuming and proficiency-demanding planning of double oblique cardiac views before their acquisition, all while the patient is lying in the scanner without acquiring data. This is reflected by the low acquisition efficiency for the reference standard, which was found to be approximately 13% in the present study. Since each operator plans these cardiac views slightly differently, an operator variability may be introduced that could potentially impact the resulting volumetric and functional measures. Furthermore, all desired cardiac views need to be decided on beforehand, as no additional views can be created afterward. In contrast, free-running CMR solves all the above challenges since no double oblique planning is required and only a 3D acquisition volume needs to be centered around the heart. Furthermore, every desired cardiac view can be required a posteriori using multiplanar reconstruction (MPR) of the 3D image (for each cardiac phase and respiratory state). This powerful feature also allows for additional measurements afterward that were not decided on beforehand, such as the assessment of atrial volumes and the coronary arteries. Using free-running MRI, cardiac cine imaging can be performed not only by those specialized or trained specifically in CMR but by every operator.

In this study, free-running cine MRI with an acquisition duration as short as 1 minute could measure left-ventricular cardiac volumes and function comparable to the reference standard. Although relatively large differences in EF of approximately 15% were noted in certain individuals (Fig. 3), these were mostly attributable to whether the most basal 2D slice was included in the volumetric assessment. Making such decisions is a well-known problem in daily clinical routine 2D cine imaging with its thick slices and potential slice gap, and can have a large impact on the resulting measures [1]. These differences were neither uniformly observed across all acquisition durations for specific subjects, nor leading to a clear under- or overestimation, and may therefore be attributed to these outliers rather than being interpreted as a shortcoming of the imaging technique. In fact, 3D free-running imaging with its 1.4 mm slice thickness and excellent MPR opportunities allows for an improved assessment of the exact valve plane, and thereby potentially more accurate assessment of both cardiac volumes and EF.

When putting the current study results in perspective, it should be noted that the inter-observer agreement analysis showed a relative difference of 8% in left-ventricular EF for standard 2D cine imaging. When comparing free-running to standard 2D cine imaging, the bias between these was smaller than the inter-observer difference for all acquisition durations. Even though the limits of agreement for 6 minutes of free-running were the smallest of all free-running acquisitions, there was no clear decrease in limits of agreement observed when acquisition duration increased. These results suggest that acquisitions as short as 1 minute already yield images that, although the image quality is significantly lower than that of conventional 2D cine, still enable accurate volumetric and functional measurements.

The present study findings, therefore, underline the importance of focusing on obtaining similar clinical measures and improving efficiency when evaluating novel MRI techniques, instead of only striving for optimal image quality. Currently, CMR examinations are long and costly, which is in part attributable to ECG setup, inefficient planning and acquisition, and repeated breath-holds. This is underlined by the low acquisition efficiency of only 13% for standard 2D cine imaging in this study. Given the recent surge in CMR, improving acquisition efficiency and reducing scan time have become more critical than ever. In this study, free-running cardiac cine MRI has demonstrated to boost acquisition efficiency by 285–560% while obtaining clinical measures similar to those derived from standard 2D cine imaging and improved ease-of-use for the operator. It should be mentioned though that cardiac view planning for free-running imaging is now performed by the clinical reader after reconstruction, instead of by the MRI operator before acquisition, and this process is therefore not incorporated in the acquisition efficiency metric for free-running imaging.

## 5 Limitations

Apart from the clear benefits in simplicity, efficiency, and patient comfort for daily clinical routine use, free-running cardiac cine MRI also has its limitations. Since a large 3D volume is continuously excited rather than just a 2D slice, free-running cine imaging cannot benefit from the inflow of undisturbed protons and thereby has reduced, albeit still sufficient, blood-myocardium contrast compared to standard 2D cine imaging. In addition, since all acquired data are retrospectively divided over both the respiratory (four motion states) and cardiac (∼20 cardiac phases) dimensions, each of the approximately 80 individual bins is heavily undersampled, making the resulting images appear inferior in quality compared to standard 2D cine imaging. This was confirmed by the qualitative assessment where standard 2D cine imaging scored significantly higher than all free-running acquisitions on both image quality and observer confidence, even though the clinical measures were similar. When desired, the image quality of the free-running data can be easily enhanced by constructing 8 mm thick slices of the acquired image data (compared to its native 1.4 mm) similarly to the 2D cine images, thereby significantly improving the SNR albeit at the expense of additional through plane blur. Finally, while boosting efficiency, simplicity and patient comfort, free-running MRI now shifts the burden toward a more complex image reconstruction that is currently performed offline and a posteriori slice selection by the clinical observer. Although current image reconstructions may take between 2 and 4 h depending on acquisition duration, more efficient code implementations and novel reconstruction methodology are expected to vastly shorten reconstruction time and finally enable image reconstruction directly at the scanner.

Future research in free-running cine CMR should focus on large multi-center validation studies with patient cohorts of various cardiomyopathies. In addition, fat suppression techniques may significantly improve image quality by reducing the residual streaking artifacts. Various solutions to implement fat suppression in free-running imaging have been proposed, including FISS [11], LIBRE [12], and HydrOptiFrame [13], which should be further investigated. Furthermore, the use of recently introduced motion fields during image reconstruction may be further investigated to improve image fidelity, thereby facilitating myocardial delineation [14], [15]. Artificial intelligence may play a pivotal role in the additional denoising of the acquired data [2], [16]. Finally, instead of using an offline image reconstruction, new reconstruction methods that enable a simplified yet fully motion-resolved static 3D image reconstruction directly at the scanner within a matter of minutes may help support broader clinical adoption of the technique [17], [18].

## 6 Conclusion

Free-running CMR with an acquisition duration as short as 1 minute can provide comparable left-ventricular cardiac volumes and EF as standard 2D breath-held cine imaging in healthy volunteers, albeit at the expense of both image quality and observer confidence. Given that free-running CMR requires no ECG, no repetitive breath-holds, no time-consuming and proficiency-demanding cardiac planning, and allows for any desired cardiac view after acquisition, and it holds promise as an alternative to standard 2D cine imaging to improve workflow efficiency, simplicity, and patient comfort.

## Funding

R.J.H. was supported by a Niels Stensen Fellowship grant. This research was in part supported by S.N.S.F. grants of R.B.v.H. (CRSII5_202276), C.W.R. (PZ00P3_202140), J.Y. (310030_215604), and M.S. (320030B_201292).

## Author contributions

**Ruud B. van Heeswijk:** Writing – review & editing, Supervision, Methodology, Funding acquisition. **Jérôme Yerly:** Writing – review & editing, Supervision, Software, Methodology, Funding acquisition. **Ludovica Romanin:** Writing – review & editing, Project administration, Investigation. **Isabel Montón Quesada:** Writing – review & editing, Software, Methodology. **Estelle Tenisch:** Formal analysis, Writing – review & editing. **Christopher W. Roy:** Software, Validation, Writing – review & editing. **Milan Prsa:** Writing – review & editing, Writing – original draft, Validation, Project administration, Methodology, Formal analysis. **Matthias Stuber:** Writing – review & editing, Supervision, Resources, Methodology, Funding acquisition, Conceptualization. **Robert J. Holtackers:** Writing – review & editing, Writing – original draft, Visualization, Project administration, Methodology, Investigation, Funding acquisition, Formal analysis, Data curation, Conceptualization. **Augustin C. Ogier:** Writing – review & editing, Writing – original draft, Software, Methodology.

## Declaration of competing interests

The authors declare the following financial interests/personal relationships which may be considered as potential competing interests. Robert J. Holtackers reports financial support was provided by Niels Stensen Fellowship. Ruud B. van Heeswijk, Christopher W. Roy, Jerome Yerly, and Matthias Stuber report financial support was provided by Swiss National Science Foundation. Ludovica Romanin reports a relationship with Siemens Healthcare that includes: employment. Matthias Stuber reports a relationship with Siemens Healthcare that includes non-financial support. Ruud B. Van Heeswijk is an associate editor of JCMR. Matthias Stuber is a senior advisor of JCMR. The other authors declare that they have no known competing financial interests or personal relationships that could have appeared to influence the work reported in this paper.
